# Individual motion perception parameters and motion sickness frequency sensitivity in fore-aft motion

**DOI:** 10.1007/s00221-021-06093-w

**Published:** 2021-03-29

**Authors:** Tugrul Irmak, Ksander N. de Winkel, Daan M. Pool, Heinrich H. Bülthoff, Riender Happee

**Affiliations:** 1 Delft University of Technology, Mekelweg 2, 2628 CD Delft South Holland, Netherlands; 2 Delft University of Technology, Kluyverweg 1, 2629 HS Delft South Holland, Netherlands; 3 Max Planck Institute for Biological Cybernetics, Max-Planck-Ring 14, 72076 Tübingen Baden-Württemberg, Germany

**Keywords:** Motion sickness, Motion perception, Frequency sensitivity, Modelling

## Abstract

Previous literature suggests a relationship between individual characteristics of motion perception and the peak frequency of motion sickness sensitivity. Here, we used well-established paradigms to relate motion perception and motion sickness on an individual level. We recruited 23 participants to complete a two-part experiment. In the first part, we determined individual velocity storage time constants from perceived rotation in response to Earth Vertical Axis Rotation (EVAR) and subjective vertical time constants from perceived tilt in response to centrifugation. The cross-over frequency for resolution of the gravito-inertial ambiguity was derived from our data using the Multi Sensory Observer Model (MSOM). In the second part of the experiment, we determined individual motion sickness frequency responses. Participants were exposed to 30-minute sinusoidal fore-aft motions at frequencies of 0.15, 0.2, 0.3, 0.4 and 0.5 Hz, with a peak amplitude of 2 m/s^2^ in five separate sessions, approximately 1 week apart. Sickness responses were recorded using both the MIsery SCale (MISC) with 30 s intervals, and the Motion Sickness Assessment Questionnaire (MSAQ) at the end of the motion exposure. The average velocity storage and subjective vertical time constants were 17.2 s (STD = 6.8 s) and 9.2 s (STD = 7.17 s). The average cross-over frequency was 0.21 Hz (STD = 0.10 Hz). At the group level, there was no significant effect of frequency on motion sickness. However, considerable individual variability was observed in frequency sensitivities, with some participants being particularly sensitive to the lowest frequencies, whereas others were most sensitive to intermediate or higher frequencies. The frequency of peak sensitivity did not correlate with the velocity storage time constant (*r* = 0.32, *p* = 0.26) or the subjective vertical time constant (*r* = − 0.37, *p* = 0.29). Our prediction of a significant correlation between cross-over frequency and frequency sensitivity was not confirmed (*r* = 0.26, *p* = 0.44). However, we did observe a strong positive correlation between the subjective vertical time constant and general motion sickness sensitivity (*r* = 0.74, *p* = 0.0006). We conclude that frequency sensitivity is best considered a property unique to the individual. This has important consequences for existing models of motion sickness, which were fitted to group averaged sensitivities. The correlation between the subjective vertical time constant and motion sickness sensitivity supports the importance of verticality perception during exposure to translational sickness stimuli.

**Supplementary Information:**

The online version contains supplementary material available at 10.1007/s00221-021-06093-w.

## Introduction

Motion sickness is a syndrome whereby aggravating body motions trigger autonomic symptoms such as salivation, dizziness, headaches, panting, hot/cold flushes, stomach awareness, nausea and vomiting (Bertolini and Straumann 2016). Chronic exposure to sickening motions may lead to the sopite syndrome, which is associated with lethargy, fatigue and drowsiness (Matsangas and Mccauley 2014).

Riccio and Stoffregen (1991) argue that motion sickness is caused by postural instability (known as postural instability theory of motion sickness). Others argue that sickness occurs due to a mismatch between sensed sensory signals and the sensory signals expected by the brain (Bos 2011) (Sensory Conflict Theory), and that postural instability is a consequence of such mismatch.

Reason (1978) was the first to promote the sensory conflict theory. Here, the predicted sensory signals were hypothesized to originate from an internal model, which takes the form of a neural store. He hypothesised that this conflict leads to adaptation of the internal model and consequently, habituation to the sickening stimuli. Oman (1982) likened this conceptual model to the manner by which a Luenberger Observer (LO) operates. The LO has an internal model of the system (which is composed of both body and environment dynamics) and sensor dynamics. Due to the imperfect and noisy nature of the sensory signals (Faisal et al. 2008; Nouri and Karmali 2018; Jamali et al. 2013) one cannot use the sensor measurements directly. Instead, the true states of the system must be observed (estimated) by integrating sensory information using an internal model of the system itself. Indeed, there is strong neuronal evidence for the use of internal modelling for state estimation (Merfeld et al. 1999; Angelaki et al. 2004; Laurens et al. 2013; Oman and Kathleen 2015). These estimated states are then used for task planning and execution. To quantify estimation accuracy, the central state estimates are passed through the internal model of sensory dynamics and compared with the actual sensory signals. The resulting error is the estimation error, or the sensory-expectancy conflict. This conflict is used to drive the estimated body motions towards the true state, and to adapt the parameters of the internal model, such that it provides better estimates. It is hypothesized that the conflict is integrated and the subsequent symptoms of motion sickness are due to its accumulation (Dai et al. 2010).

Therefore, based on the strong neuronal support for internal modelling, the scope of this paper will not cover postural instability theory nor attempt to evaluate postural precursors to motion sickness, instead, we aim to build on the concepts of state estimation and sensory conflict.

The form this state estimation model needs to take to make accurate predictions of motion sickness is not clear. If the human state estimation process is indeed linked to motion sickness, one would expect to see a clear relationship between certain parameters of state estimation and motion sickness. State estimation is a latent process and cannot be directly measured. Instead, one may measure its correlates in reflexive actions, such as eye movements elicited by the vestibular-ocular reflex (VOR) or through perceived motions such as angular velocity, linear displacement and orientation, as reported subjectively by human participants (Merfeld et al. 2005). From such correlates, simple individual-specific parameters can be derived such as the velocity storage time constant and the subjective vertical time constant. These parameters characterize motion perception and reveal key aspects of an individual’s state estimation process. Here, the velocity storage time constant is indicative of how the individual computes ego-angular velocity (Bertolini et al. 2011) and the subjective vertical time constant provides a measure of the frequency characteristics of tilt and translation perception.

Several studies investigated the relationship between individual motion perception parameters and motion sickness susceptibility. Dai et al. (2003, 2007) used a cross-coupled motion paradigm where participants were requested to roll their head about the naso-occipital axis whilst being rotated in yaw. The number of head rotations a participant could tolerate was negatively correlated with the velocity storage time constant (*r* $=$ $-0.94$). In addition, as the participants followed a habituation protocol, there was a significant reduction in the velocity storage time constant, in tandem with an increase in the number of tolerable head movements (Dai et al. 2003).

Relatedly, Clément et al. (2001) used the cross-coupled motion paradigm, this time with pitching motion about the interaural axis of the head. They found that during habituation to the sickening motion, there was again a significant decrease in the duration of the post-rotary nystagmus (and consequently in the velocity storage time constant), as well as in motion sickness. In a similar experiment, Clément et al. (2007) found that the velocity storage time constant reduced by 22.7% with habituation.

Though there seems to be converging evidence of a strong relationship between the velocity storage time constant and motion sickness sensitivity, Quarck et al. (2000) found no significant differences between the velocity storage time constant of sickness susceptible and non-susceptible participants. Likewise, Tanguy et al. (2008) found no difference in the velocity storage time constant between figure skaters (who were significantly less susceptible to motion sickness) and controls.

It is important to note that the nauseogenic stimulus in both Quarck et al. (2000) and Tanguy et al. (2008) was off-vertical axis rotation (OVAR), which is different from the cross-coupled stimulation employed in the former studies. Here, as during constant rotation about the body axis, the semi-circular canal stimulation reduces to zero. The effect of angular velocity perception on sickness sensitivity should therefore be much lower in this motion paradigm.

The potential cause of sickness in OVAR was investigated by Wood (2002). Here, it was noted that the modulation of the torsional and horizontal components of eye velocity, indicative of tilt and translation, respectively, crossed over at approximately the region where Denise et al. (1996) measured peak sickness during OVAR, which was approximately at 0.3 Hz for their group of participants. Following on from this study, Wood et al. (2007) measured tilt and translation perception above and below the previously identified cross-over frequency. The cross-over frequency characterises how the central nervous system performs gravito-inertial ambiguity resolution (referred to in some literature as tilt-translation ambiguity resolution). The ambiguity resolution is necessary because the otoliths do not measure inertial acceleration separately from gravity. Instead, due to Einstein’s equivalence principle, these are sensed in the form of a combined vector named gravito-inertial acceleration (or specific force). For appropriate actuation of an organisms’ effectors, this combined vector must be decomposed into acceleration and gravity. For the OVAR motion paradigm, the central nervous system attempts gravito-inertial ambiguity resolution by employing an internal model which can be effectively seen as high (for translation) and low-pass (for tilt) filtering of the otolith signal. The time constant for tilt perception (referred to as the subjective vertical time constant), determines the cross-over frequency and has been hypothesized to correlate with the peak frequency of sickness (Wood 2002).

Correlating a single parameter with sickness sensitivity for a single motion paradigm, as in the angular vestibular-ocular reflex (aVOR) time constant studies, may provide misleading conclusions of a general sensitivity effect of this parameter, rather than for example the frequency effect that was found by Wood (2002), Dai et al. (2007), Denise et al. (1996) for OVAR.

From a system identification perspective, the mechanistic relationship between motion perception parameters and sickness, such as through the observer framework as used by Oman (1982) and Bos and Bles (1998), only becomes identifiable when the frequency sensitivity of sickness is measured and correlated with the parameters. Bos and Bles (1998) and Khalid et al. (2011) attempt to identify such a model of motion sickness by fitting mean perception parameters from literature to group level frequency sensitivity data collected for vertical (O’Hanlon 1974) and lateral accelerations (Donohew and Griffin 2004), respectively. Individuals, however, differ largely from each other in their motion sickness sensitivity (Lackner 2014) and likely also differ in their frequency sensitivity to sickness. If this is the case, group average frequency sensitivity may not be the same as individual frequency sensitivity, which may vary substantially between individuals, therefore using group average sensitivity to fit these models may not be correct.

In the present study, we will assess individual variability in the frequency response of motion sickness sensitivity and evaluate the relation with motion perception parameters. We will do this by having participants perform a two-part experiment. In the first part, we will determine the time constant of velocity storage and the subjective vertical for each individual. The former parameter will be evaluated by using an Earth Vertical Axis Rotation (EVAR) motion paradigm. The latter will be evaluated both by computing the time constant of subjective tilt change during centrifugation (not taking into account the effect of yaw present during centrifugation), as well as in a more nuanced manner, by computing the cross-over frequency using the Multi Sensory Observer Model (MSOM) (Newman 2009; Newman et al. 2012; Clark et al. 2019). The motions will be generated using the Cyber Motion Simulator at the Max Planck Institute for Biological Cybernetics in Tübingen (Barnett-Cowan et al. 2012). In the second part of the experiment, we will determine frequency sensitivity to horizontal plane accelerations, just as in Donohew and Griffin (2004), Golding and Markey (1996) and Golding et al. (1997), but specifically evaluating the response at the individual level. This will be done on the Max Planck CableRobot simulator (Miermeister et al. 2016). In subsequent analyses, we will relate the perception parameters obtained in the first part of the experiment to the sickness response observed in the second part of the experiment. Based on the literature, we hypothesize that:

1. Group sensitivity of sickness due to fore-aft acceleration is frequency dependent.
2. Frequency-dependent sickness sensitivity varies substantially between individuals.
3. Individual motion perception parameters are indicators of motion sickness sensitivity:
  a The peak frequency of sickness is expected to increase with lower subjective vertical time constant and higher cross-over frequency;
  b The peak frequency of sickness is expected to correlate with the velocity storage time constant;
  c Overall motion sickness sensitivity is expected to correlate with the subjective vertical time constant, cross-over frequency and the velocity storage time constant.

## Methods

The study was designed to assess whether a relation exists between parameters that describe perception of passive self-motion from vestibular stimulation and motion sickness sensitivity. To this end, we performed a combined set of two experiments, on 1) motion perception and 2) motion sickness sensitivity as a function of frequency.

### Ethics statement

All participants provided written informed consent prior to participation. The experimental protocol was approved by the ethical committee of the Human Research Ethics Committee of TU Delft, The Netherlands, under application number 1030.

### Participants

In total, 23 participants took part in this study (mean age: 26.7 years, STD: 4.1 years, 15 female, 8 male). Participants were compensated for their time at a rate of 10 €/hr, with a 30€ bonus upon completion of all 6 sessions. Due to the incipience of the COVID-19 pandemic (2020), we were forced to stop data collection abruptly. As a consequence, 12 participants completed all 5 sickness sessions; 6 completed 4; 3 completed 3; and 1 completed only 2 sessions. The 23 participants had a mean motion sickness susceptibility questionnaire short form (MSSQ-Short Golding (2006)) score of 16.6 (STD = 10.5), indicating an above average sensitivity, corresponding to the 67th percentile. The MSSQ-Short scores were not used in the participant selection process.

### Apparatus

For both experiments a neck-brace was used to limit unwanted head rotations. Participants were also blindfolded by the placement of a hollowed out VR headset over their eyes. A Tobii Pro glasses eye-tracker (Tobii Pro AB, Danderyd, Stockholm, Sweden) was placed within the VR headset.

#### Perception experiment

**Fig. 1:**
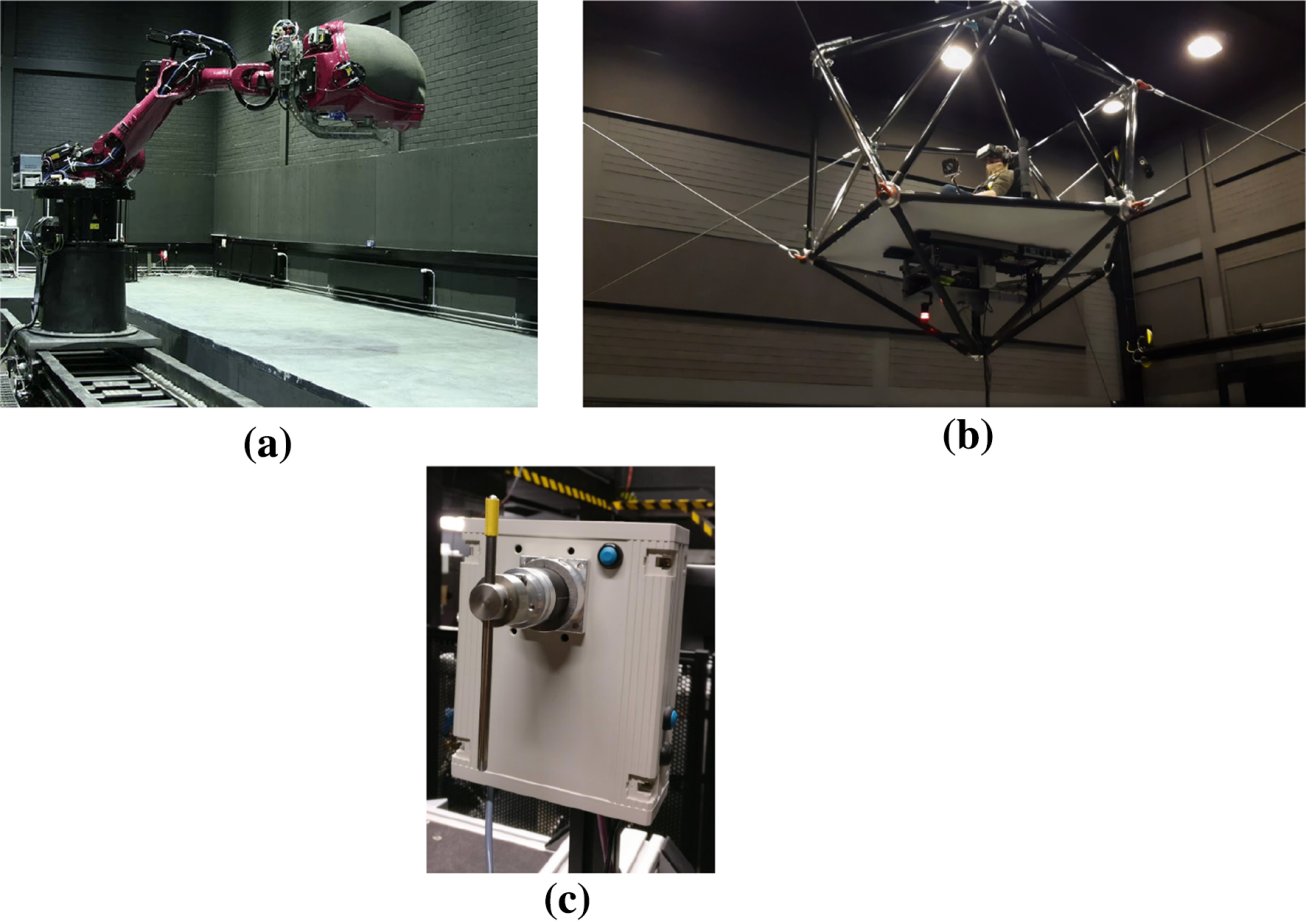
Experimental setup. **a** is the CMS used to measure motion perception in centrifugation mode. **b** shows the CRS used to measure frequency sensitivity in motion sickness development. **c** shows the pointer device, participants were instructed to align the orientation of the rod to (the pitch of) their perceived vertical

This experiment was performed using the Max Planck Institute’s CyberMotion Simulator (CMS, see Fig. 1a) (Barnett-Cowan et al. 2012). The CMS is an anthropomorphic robot arm (KUKA Roboter GmbH, Augsburg, Germany, model Robocoaster) at the end of which is a closed cabin within which the participant is seated and secured using a five point harness. During the experiment, participants wore ear-enclosing headphones that attenuated the rumble noise of the simulator. Continuous communication with the experimenter could be made via the integrated microphone. Perceptual responses were provided by the participant using a custom made pointer device. The pointer device (see Fig. 1c) consists of a stainless steel rod of approximately 20 cm that is connected through the middle to a rotary potentiometer. The potentiometer is housed in a plastic box that was placed above the right leg of the participant such that it may be actuated by the right hand. The rod could be rotated in the vertical sagittal plane.

#### Sickness experiment

Motion sickness was elicited by fore-aft motions performed on the Max Planck Institute’s CableRobot Simulator (CRS, see Fig. 1b) (Miermeister et al. 2016). The CRS is a cable driven simulator: the cables are attached to a central cabin and actuated by electric motors controlling the extension of the cables. The allowable work space of the cabin is 8 m$\times$4 m$\times$4 m (longitudinal, lateral and vertical in the seating direction). The participant was seated in a racing chair and secured via a five-point harness. An additional safety belt was placed across the lap of the participant. During the experiment, participants wore ear-enclosing headphones with an embedded microphone, which allowed continuous communication. Sickness ratings were queried in 30 s intervals with a 1 kHz beep and verbal responses were recorded automatically to a computer connected to the microphone.

### Task and stimuli

#### Perception experiment

All participants first took part in the perception experiments. The CMS was used to provide stimuli to estimate motion perception parameters of the velocity storage time constant and the subjective vertical time constant.

Centrifugation was used to determine the subjective vertical time constant. The centrifuge arm was rotated with an angular acceleration as a function of time, *t* of the form: $47.5(-\text {cos}(2\pi ft) + 1)$ deg/s^2^, where $f = 0.709$. This lasted for 1.41 s, at which point the centrifuge reached a constant earth vertical rotation velocity of 67 deg/s. Participants were placed with a radial offset of 3.11 m from the centre of centrifuge rotation. They were oriented facing out from the direction of centripetal acceleration. Participants were instructed to align the orientation of the rod to (the pitch of) their perceived vertical (Correia Grácio et al. 2013). As the participants were blindfolded, this was done based on the sense of touch only.

Velocity storage time constants were determined in conditions where the CMS was used to rotate participants around an Earth-vertical axis (EVAR) at 57 deg/s The angular acceleration was a function of time, *t* of the form: $47.5(-\text {cos}(2\pi ft) + 1)$ deg/s^2^, where $f = 0.833$. This lasted for 1.2 s, at which point the rotation reached a velocity of 57 deg/s. Here, participants were instructed to indicate their perceived angular velocity by rotating the rod away from or towards their body, in proportion to the subjectively experienced speed and the direction of their perceived rotation, the displacement of the rod from the vertical for EVAR indicated the speed of the perceived angular velocity, with the anchoring point being the upright position, indicating standstill (the rod was reset to this position for every new motion trial). This psychophysical task is similar to the one used by Bertolini et al. (2011), who instructed their participants to match their perceived speed with the speed of rotation of a similar rod. In our experiment, participants were instead asked to do the easier control task of matching their perceived speed, with the position of the rod. The mapping of rod deflection to spin rate is arbitrary, so we cannot know what the actual perceived speed of the participant was. However, the responses do indicate how the perceived speed decayed with respect to time, which is the only requirement for determining the velocity storage time constant. Therefore, just as in Bertolini et al. (2011), the amplitude of rod deflection was normalized. The normalization was done with respect to the maximum rod position during the initial 60 s rotation.

The two motion paradigms were each repeated 4 times for a total of 8 trials. The motion paradigms from the 8 trials were presented in random order. Including the fade-in/fade-out periods, the motions for each trial lasted for 60 s. After this, the platform was stopped and remained stationary for another 60 s. Participants were instructed to report their perceptions also during the stationary phase. After the stationary phase, the platform was re-orientated for the next trial.

Vertical and horizontal eye movements were recorded using the Tobii Pro glasses and processed, but not analysed further as the data was of poor quality in approximately 50% of the participants.

#### Sickness experiment

The week after the perception experiments on the CMS, participants began their first motion sickness session. Each participant underwent five motion sickness sessions, separated by a minimum of 4 days to minimise habituation. Participants were seated on the CRS platform and subjected to (maximum) 30-minute sinusoidal fore-aft motions with peak accelerations of 2 m/s^2^ and frequencies of 0.15, 0.2, 0.3, 0.4 and 0.5 Hz, with a 10-second smoothed start and end. The fade-in function for time *t* was: $(t/10) - (1/2\pi ) \sin (2\pi (t/10))$. For the fade-out, the value of this function was subtracted from 1.

Each session only tested one frequency of the acceleration stimulus. Due to time limitations and a desire to sample as broad a range of frequencies as possible, conditions were not repeated. This is justified by good trial-to-trial repeatability found in motion sickness response (Miller and Graybiel 1969; Irmak et al. 2020). The choice of the lowest frequency was imposed by the maximum possible stroke at 2 m/s^2^. The choice for the highest frequency was based on the observed frequency at which sickness was approximately one tenth of that at lower frequencies (Donohew and Griffin 2004). The order in which each frequency was experienced was randomized for all the participants. This prevented confounding effects of habituation between the different frequencies.

The participants were instructed to report their sickness on the 11-point subjective MIsery SCale (MISC) (Bos et al. 2010). Although MISC may probe multiple symptom clusters, MISC is still monotone with respect to subjective severity of sickness. This is supported by the fact that in our study mild nausea (MISC 6) was never seen prior to build up of the other symptoms (Fig. 12 in the Appendix), an observation that has also been reported in previous literature (Bos et al. 2005). Therefore, the scores recorded are on an ordinal scale with respect to sickness severity. The MISC was queried every 30 seconds. Each sickness session lasted up to a maximum of 30 minutes, or until the participant reached a MISC level of 6. A MISC level of 6 corresponds to slight nausea and was deemed an appropriate threshold both in order to prevent participant dropout from further sessions but also due to ethical concerns. After each session, participants were asked to complete the Motion Sickness Assessment Questionnaire (MSAQ) (Gianaros et al. 2001). The MSAQ is a 16-item questionnaire composed of 4 sub-components querying various types of sickness. The types of symptoms are: gastrointestinal symptoms such as stomach awareness, central symptoms such as feeling light headed, peripheral symptoms such as feeling sweaty, and sopite symptoms such as fatigue. Each item was rated on a scale from 1 to 9, providing Likert-type ordinal data. The MSAQ gave a more detailed breakdown of participant symptoms at the worst point of their sickness, which was almost always at the end of the experimental session.

### Data reduction

A large amount of data was collected in both parts of the experiment. To evaluate our hypotheses, we summarized the perception data to a simple set of parameters that characterized each participants’ response. Likewise, for each sickness session we calculated a single robust metric that represented the amplitude of the sickness experienced.

#### Velocity storage and subjective vertical time constants

The subjective vertical and velocity storage time constants for perception were identified by first pooling the normalized responses of each motion paradigm per participant and averaging across the repetitions. The time constants of the perceived subjective vertical and the angular velocity were estimated as the time it took for the perceived quantity to converge 63.2% of the way to steady state. For the subjective vertical this was estimated by fitting an exponential function of the following form $y = b_1(1-exp(-b_2t))$ using the *fitnlm* function in MATLAB. The average $R^2$ value across individuals was 0.96, indicating a good fit of the model (individual fitting shown in Fig. 9a in the Appendix). For velocity storage, the time constant was estimated non-parametrically by simply finding the point at which the perceived angular velocity passed 63.2% of the way to steady state (individual responses as shown in Fig. 9b in the Appendix). A different method than fitting an exponential was chosen here because of plateauing behaviour. This is discussed in the results.

#### Cross-over frequency

The subjective vertical time constant derived from the centrifugation is specific to the experimental paradigm used. If a variable arm centrifugation would have been used, or a faster yaw velocity, the time constant of the subjective tilt would likely be different. This is due to the presence of the perceived yaw signal (Merfeld et al. 2000), which is influenced by the velocity storage time constant. The fore-aft acceleration that was used to induce sickness does not have this angular component. For this perturbation, just as in OVAR, it is likely that the cross-over frequency of gravito-inertial perception is the underlying parameter of most importance. The perception paradigms used do not allow for a direct measurement of this value. However, the results from the paradigms studied can be used with models of sensory integration to estimate the cross-over frequency of gravito-inertial perception. For this purpose we use the Multi Sensory Observer Model (MSOM) (Newman 2009; Newman et al. 2012; Clark et al. 2019). The MSOM was chosen because it has been validated across a wide range of motion paradigms and belongs to the observer class of spatial orientation models linked by Oman (1982) to motion sickness development.

**Fig. 2:**
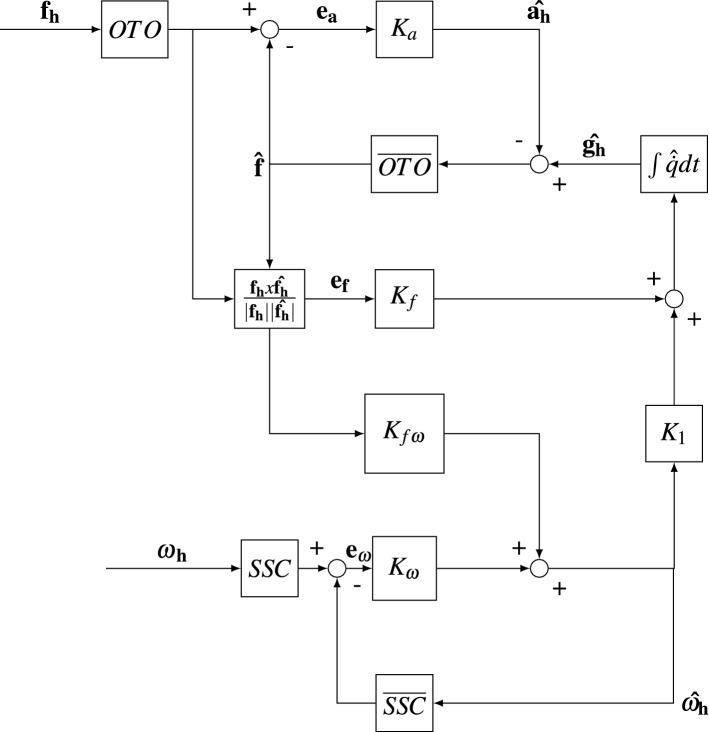
Multi Sensory Observer Model used to compute the cross-over frequency between acceleration and gravity perception

The MSOM, shown in Fig. 2, was implemented in Simulink. The aim of the MSOM is to predict perceived angular velocity, inertial acceleration and orientation of the human participant given the inputs of head referenced head angular velocity $\omega _{h}$ and head referenced gravito-inertial force $f_h$. In this model perceived orientation and so the the rod orientation set by the participant is assumed to be inline with the gravitational vector. This assumption for passive centrifugation matches experimental observations (Panic et al. 2015; de Winkel et al. 2020), such as in this study, but may not hold for active motion and control of orientation, where the upright may be defined with the direction of balance instead (Riccio et al. 1992). In its current formulation the MSOM has five parameters, $K_{\omega }$, $K_{f\omega }$, $K_f$ and $K_a$ and $K_1$. The parameter $K_{\omega }$ is the feedback gain of the angular velocity perception path. For a given time constant of the semi-circular canals (*SSC*) and the internal model of the semi-circular canals ($\overline{SSC}$) its value determines the velocity storage time constant of angular velocity perception. The otoliths (*OTO*) and the internal model of the otoliths ($\overline{OTO}$) is given as a unity transfer function. The parameter $K_{f\omega }$ determines the contribution of a change in the orientation of the gravito-inertial force on the perceived angular velocity. Parameter $K_f$ sets the change in perceived orientation given a change in the orientation of the gravito-inertial force. Parameter $K_a$ is the translational acceleration feedback gain, determining the gain of the perceived acceleration through the otolith organs. Lastly, $K_1$ is the angular velocity gain to the gravity estimator. This is actually a function of $K_\omega$ of the form $K_{\omega }/(1 + K_{\omega })$

For our implementation, the gain $K_{f\omega }$ was set to zero because it was found that during OVAR perception of angular velocity tended to zero, which would not be the case for a finite $K_{f\omega }$ (Vingerhoets et al. 2005). Moreover, the semi-circular canals *SSC* and the internal model of the semi-circular canals $\overline{SSC}$ were both represented as first-order high-pass systems, with a time constant fixed at 5.7 s for all participants based on Merfeld et al. (1993). Setting this as a free variable would have created an underdetermined model. The reference data for model fitting was the rod setting for the centrifugation and the EVAR motion paradigms. Because the model is not able to predict biases, such as an overshoot of the subjective vertical or miscalibration in the internal representation of rod orientation, as may have been the case for some of the participants, the rod data was first filtered through a median filter of window 1 s, it was then normalised and scaled to the theoretically expected maximal tilt and angular velocity percepts. This operation did not affect the predicted cross-over frequency, as even though amplitude information was lost, the temporal dynamics were maintained.

For reference, Fig. 10 in the Appendix shows the fits for the 14 participants for which both centrifugation and EVAR data was available. The fits were performed using a genetic algorithm with a group size of 100 for each generation. The fitting was then further refined using the interior-point method. This process was repeated 5 times from different initial conditions. The cross-over frequencies found were approximately the same between different iterations and were therefore averaged across iterations per participant. The error function used for the fitting was The Symmetric Mean Absolute Percentage Error (SMAPE):

$$\begin{aligned} \text {SMAPE} = 100\frac{\varSigma ^n_{t=1}|F_t - A_t|}{\varSigma ^n_{t=1}|A_t| + |F_t|} \end{aligned}$$

This error metric is well protected against outliers and treats both over and underestimation in an unbiased manner. For each iteration, the SMAPE between the fitted, $F_t$ and the actual $A_t$ perceived angular velocity and subjective vertical were calculated. The average of the two errors was then minimised by the optimisation.

**Fig. 3:**
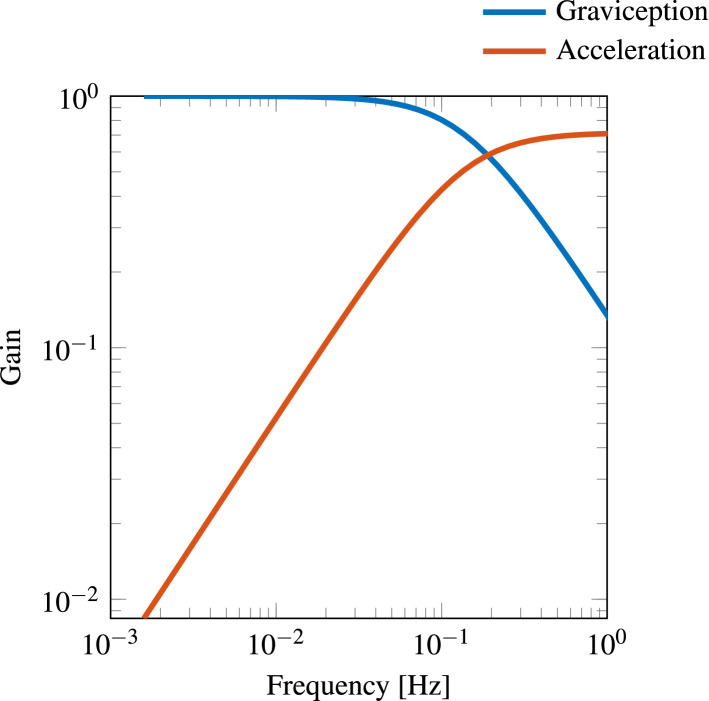
Example of one participant showing a cross-over frequency of 0.18 Hz

The solution for $K_{\omega }$ was unique for each individual. The parameters $K_a$ and $K_f$, however, could not be uniquely estimated from the current data. However, the ratio between the $K_a$ and $K_f$ values found was unique. It is this ratio that determines the cross-over frequency, which is given by the intersection of acceleration and gravity perception gains, as shown in Fig. 3 (and Fig. 11 in the Appendix for all participants). Figure 3 was generated by plotting the magnitude response of the linearised simulink model (obtained using bode response option native to Simulink). This subsequent linearisation gave as outputs gravity and acceleration for a small horizontal acceleration input perturbation. The factor by which this small horizontal acceleration becomes gravity and acceleration respectively is given as the gain. In this case Fig. 3 shows the small acceleration perturbation at lower frequencies is perceived as change in gravity, i.e. tilt and at higher frequencies as acceleration. Therefore, it was possible to use our two motion perception paradigms to determine a unique cross-over frequency as per the MSOM.

#### Sickness magnitude

In this study, motion sickness was measured using two methods: the MISC ratings that were taken during a session, and the MSAQ that was filled out at the end of a session. MISC ratings were obtained for the duration of each sickness-session, with intervals of 30 s (Fig. 12 in the Appendix shows MISC trajectories for all participants and conditions). Sickness intensity can be quantified in different ways, for example, using the mean MISC, which is the average MISC rating across the entire period of the run, or the MISC rate, which is the maximum MISC rating divided by the time taken to reach it. Likewise, for the MSAQ, either the total score or the various sub-scores can be used to quantify the frequency sensitivity. Each measure can transform the frequency sensitivity curves seen, with no guarantee that the chosen measure is be the best measure.

Therefore, instead of choosing a single sickness metric among the different choices in an arbitrary manner, we performed factor analysis to ascertain the latent factor structure. With the factor loadings we then established an aggregated measure that best correlated to the underlying sickness experienced by the participants. This has the added advantage of reducing measurement noise. Here, there are 7 metrics to quantify sickness: the mean MISC ($M_{\mu }$), MISC rate ($\dot{M}$), maximum MISC ($M_{\text{max}}$), MSAQ Gastro ($MS_g$), MSAQ Central ($MS_c$), MSAQ Peripheral ($MS_p$) and MSAQ Sopite ($MS_s$). These are strongly correlated with each other, but also to a number of latent factors. In our experiment we had a within-participant design where the same participant was exposed to 5 conditions in terms of motion frequencies. Hence variance is influenced by both within and between participant sources.

These metrics were first standardized with respect to their group mean and standard deviation such that they were unitless. To account for dependency in the metrics, factor analysis on the averaged within-participant correlation matrix (Reise et al. 2005) was performed (Table 1). As the data is ordinal Spearman’s rank correlation matrix was used (Klinke and Wagner 2008).

**Table 1:** Table of factor loadings of different subjective sickness scores; mean MISC, MISC rate, maximum MISC, MSAQ gastrointestinal, MSAQ central, MSAQ peripheral and MSAQ sopite, on to three latent factors

Item	Factor 1	Factor 2	Factor 3
$M_{\mu }$	**0.8984**	0.2000	0.1160
$\dot{M}$	0.1306	** 0.9884**	0.0318
$M_{\text{max}}$	**0.8816**	0.0174	0.2572
$MS_g$	**0.7897**	0.0083	0.2329
$MS_c$	0.4338	0.0444	0.4029
$MS_p$	**0.6631**	0.1257	0.0912
$MS_s$	0.1516	0.0194	**0.8512**

Items with unique loadings for a given factor are boldened

Factor 1 explained 41.1% of the variance and mainly related to the overall level of sickness, as is seen from the high loadings on the mean and maximum MISC, as well as the gastric component of the MSAQ. Factor 2 explained an additional 14.8% of the variance and mainly related to the duration of symptom development, with the highest loading for the MISC rate. Lastly, Factor 3 explained an additional 14.7% of the variance and primarily captured other sources of discomfort, such as drowsiness and irritation. This was indicated by the high loading on the sopite component of the MSAQ. As we are interested in sickness, Factor 1 is the most important. Here, the loadings on the mean MISC, maximum MISC and MSAQ Gastro and MSAQ Peripheral were greater than the loadings on the other metrics. Moreover, unlike the other items loaded on Factor 1, they did not cross-load onto the other factors. Therefore, using these metrics, a joint Sickness Index (SI) was constructed (Wieland et al. 2017). The SI is given in the form

$$\begin{aligned} SI = \frac{F_{11}}{4\sigma _1}M_{\mu } + \frac{F_{13}}{4\sigma _3}M_{\text{max}} + \frac{F_{14}}{4\sigma _4}MS_g + \frac{F_{16}}{4\sigma _6} \end{aligned}$$

where $F_{11}$ is the factor loading of the first factor on the first metric $M_{\mu }$, $F_{13}$ is the factor loading of the first factor on the third metric $M_{\text{max}}$ and $F_{14}$ is the factor loading of the first factor on the fourth metric $MS_g$ and lastly $F_{16}$ is the factor loading of the first factor on the sixth metric $MS_p$. Likewise, $\sigma _1$ is the standard deviation in the scores of the first item rating $M_{\mu }$, $\sigma _3$ is the standard deviation in the scores of the third item rating $M_{\text{max}}$, $\sigma _4$ is the standard deviation in the scores of the fourth item rating $MS_g$ and lastly $\sigma _6$ is the standard deviation in the scores of the sixth item rating $MS_p$. The coefficients of both $M_{\mu }$, $M_{\text{max}}$, $MS_g$ and $MS_p$ are all standardized with respect to their respective standard deviations (Fernando et al. 2012).

### Peak frequency of sickness

Due to being a more robust measure than the unprocessed peak or the one estimated in the statistical model, the spectral centroid was taken as the point of peak sickness. This was given by the equation

$$\begin{aligned} \frac{\varSigma ^N_1 f(n)SI(n)}{\varSigma ^N_1 SI(n)} \end{aligned}$$

where *N* is the nth condition for a given participant, *f* is the frequency of this condition and *SI* is the sickness index seen at this condition.

#### General Motion Sickness Sensitivity

The estimated frequency sensitivities may be affected by noise in individuals’ responses, for instance due to difficulty distinguishing between low levels of sickness, or due to day-to-day variability in sensitivity between sessions. A more robust measure of overall sensitivity to sickening stimuli was obtained by averaging the responses for different frequencies. To evaluate overall sensitivity, we calculated a mean SI across all frequency conditions available for each individual participant.

## Results

### Perception experiment

**Fig. 4:**
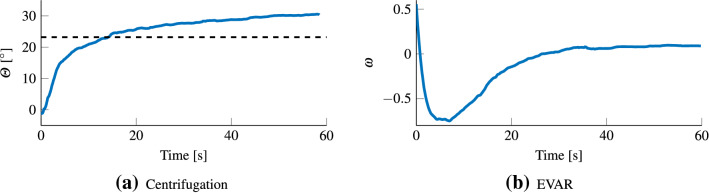
Mean perceptual responses of all participants. **a** is the mean subjective vertical tilt. The y-axis indicates the orientation of the pointer device which was taken as proxy for subjective vertical tilt. The black dashed line signifies the expected tilt. **b** shows the mean subjective angular velocity after rotation, where the y-axis indicates the normalized perceived angular velocity

**Fig. 5:**
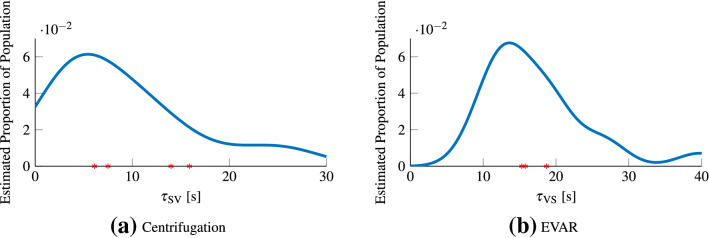
Estimated distribution of perception parameters using kernel density estimation, as well as the mean parameters findings in the literature indicated by asterisks on the x-axis. **a** estimated distribution for the time constant of the subjective vertical. The stars going from left to right are Curthoys (1996), Graaf et al. (1996), Merfeld et al. (2000) and Graybiel and Brown (1951). **b** estimated distribution for the time constant of velocity storage. The stars going from left to right are Guedry (1974), Okada et al. (1999) and Vingerhoets et al. (2005)

The centripetal force of 4.2 m/s^2^ should change the direction of the gravito-inertial force (GIF) to be 23.2 deg from the direction of gravity. Figure 4a shows the average response pooled across all repetitions for all participants. Here, there was an over-estimation (when compared to the GIF) of the final tilt angle, at 31.3 deg. The mean time constant of this tilt percept was 9.2 s (STD = 7.17 s). As shown in Fig. 5a, this time constant was similar to reported values in literature (Graybiel and Brown 1951; Merfeld et al. 2000; Curthoys 1996; Graaf et al. 1996). Figure 9 in the Appendix, shows the development of the tilt percept for all 17 participants that data could be collected for. Eight of the participants converged to within 2 deg of the GIF, another 8 showed a higher tilt angle with only one noting a lower tilt angle.

In the first 60 s of EVAR, participants reported a constant perception of rotation (shown in Fig. 9 in the Appendix). This is contrary to the expectation of a steadily decaying perception of angular velocity. This retention of perceived angular velocity was likely due to noise and rumble of the simulator. For this reason, the decay in the perceived angular velocity following a period of 60 s after the complete cessation of the simulator was used instead to quantify the velocity storage time constant. Figure 4b shows this perception after-effect for EVAR, averaged across all participants, for all repetitions. Here, the mean time constant for the decay was 17.2 s (STD = 6.8 s). As shown in Fig. 5b, this was similar to the values reported in literature (Guedry 1974; Okada et al. 1999; Vingerhoets et al. 2005). Once at the maximal value, the mean response showed a plateau of approximately 4 s. Looking at the individual responses in Fig. 9 (in the Appendix), it can be seen that for 11 participants the duration for which participants stayed at >90% of maximum tilt sensation was more than 5 s. For the remaining 7, perceived velocity decreased faster, i.e., <90% within 5 s.

### Sickness experiment

#### Group frequency sensitivity

**Fig. 6:**
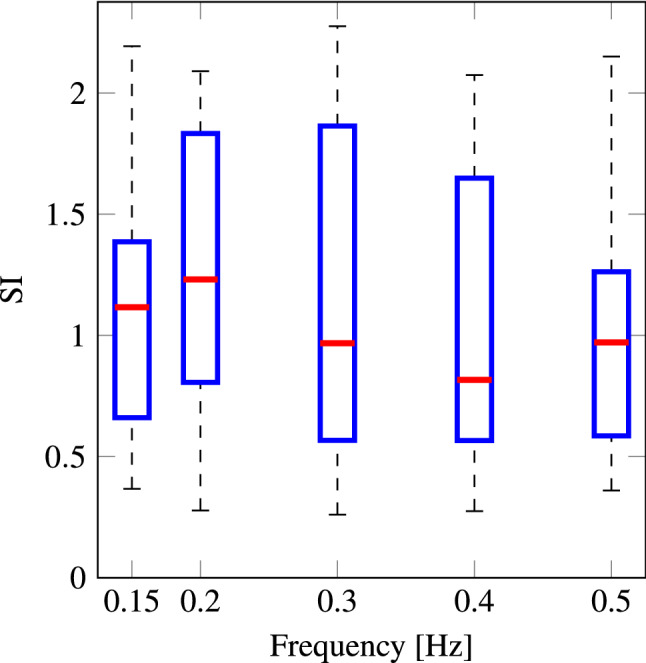
Sickness Index for different frequencies. The bars show the median, the 25th and 75th percentiles, and the full range

**Fig. 7:**
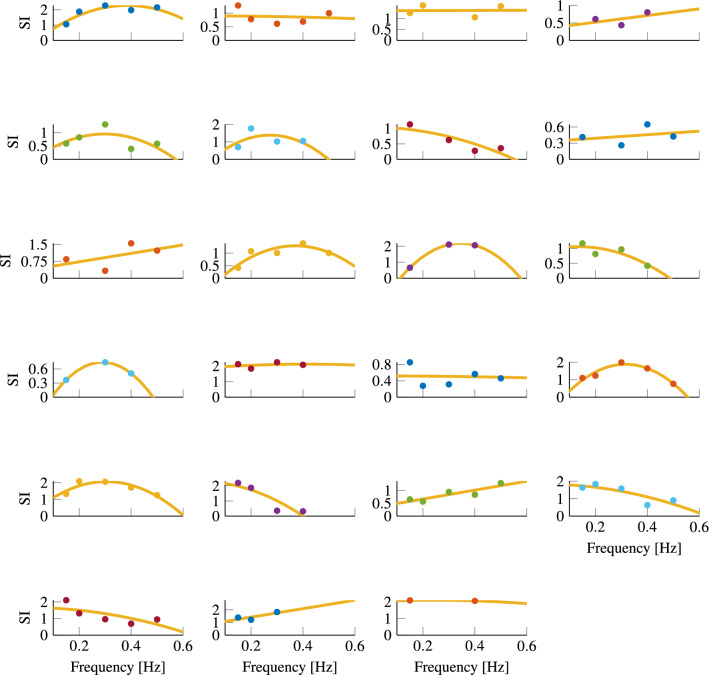
Participant sickness index as a function of frequency, with individual quadratic fits

In our study only 7 out of 23 participants reached mild nausea (MISC of 6 at one or more frequencies). This indicates a mild level of sickness was reached in this study. The Sickness Index (SI) between participants does not show a clear frequency dependency (Fig. 6). However, individual participants do show a marked frequency dependency (Fig. 7).

As per Donohew and Griffin (2004), we expected (H1), *Group sensitivity of sickness due to fore-aft acceleration to be frequency dependent*), where the relation between frequency and motion sickness reported previously can be approximated by an upside down parabola. Consequently, we assumed the sickness index to be described by a quadratic function of the form:

$$\begin{aligned} SI = b_1 - b_2(f - b_3)^2 \end{aligned}$$

where $b_1$ is the intercept, $b_2$ is the strength of the quadratic effect and $b_3$ is the frequency of maximum sickness. The coefficients $b_2$ and $b_3$ were bounded such that they were both greater than or equal to zero.

The hypothesis that there is such a quadratic relation can be tested statistically by comparing AIC and BIC scores for this model to those metrics for an intercept-only model. AIC and BIC scores are measures of model fit which are based on the likelihood of the data given the model, whilst including a penalty term for the number of parameters. This penalty term has a constant scaling with the number of parameters for the AIC, but has a logarithmic scaling with the number of observations for the BIC. This means that for a large number of observations, the BIC is more conservative in its selection of more complex models. Fabozzi et al. (2014) specifies how to interpret the absolute value of differences in the AIC and BIC scores between the models, in terms of strength of evidence. According to these rules of thumb, absolute differences in the indices $>2$, $>6$, and $>10$ provide positive, strong, and decisive evidence (respectively) in favor of the model with the lower value.

As a benchmark, we first fitted a Fixed Effect Intercept only model, $SI = b_1$. The observed AIC and BIC values were 173.5 and 176.0, respectively. For the full Fixed Effect Quadratic model, $SI = b_1 - b_2(f - b_3)^2$, the observed AIC and BIC were 174.0 and 181.6, respectively. This suggested that there was no common frequency effect in the group. Indeed, a visual inspection of the box plot in Fig. 6 is consistent with this assessment. However, when the error between model predictions and the observations made for each individual participant was evaluated, it was seen that the errors were not evenly distributed around zero, but instead, these were offset in either direction of zero (shown in Fig. 13 in the Appendix). This suggests that accounting for individual variability could improve the model fit. We attempted to account for this variability between individuals by using a mixed-effects intercept and quadratic models. However, these did not provide successful fits to the data and so for the sake of brevity this is not shown.

#### Individual sensitivity

The reason behind the frequency-invariant group-level response could be the aggregation of individual differences in frequency sensitivity. As shown in Fig. 7 individuals show markedly different sensitivity variations with frequency: for some, there appears to be a peak at intermediate frequencies (i.e., going from left to right, participants 5, 10, 13, 16 and 17), whereas others appear to experience more sickness at low frequencies (i.e., participants 7, 12, 18, 20 and 21) or at higher frequencies (i.e., participants 1, 10, 11 and 19). Also, for some participants the range of frequencies tested might not be wide enough to reveal a peak sickness response.

We tested whether individuals on average had different frequency sensitivities by fitting the model to each individual’s data separately. However, individual participants contributed at most five data points, and the model has three parameters, this left few degrees of freedom. The model could not be fitted for participants who had less than four data points, and hence, the five individuals for whom this was the case were excluded from this analysis.

Because participants are independent, we can sum model likelihoods, the number of parameters, and the number of observations to calculate overall model fit indices for the intercept-only and the quadratic models. For the intercept-only model, the AIC and BIC scores were 82.4 and 125.7, respectively, with 31.0 and 160.9 for the quadratic model. In this analysis, the AIC thus favors the quadratic model, whereas the BIC favors the intercept-only model due to a larger penalty on the number of parameters. Likelihood ratio test between the Individual Intercept and Individual Quadratic model gave $p < 0.001$, this indicates that the Individual Quadratic model is a better fit to the data.

Because this analysis excluded some participants, new joint fixed intercept and quadratic models were fitted as benchmarks to assess the evidence for individual differences. These models were fitted to the joint data of all participants included in the individual fits. The scores obtained were AIC: 144.2 and BIC: 146.6 for the intercept model and AIC: 144.0 and BIC: 151.2 for the quadratic model. The conclusions thus differ depending on the choice of criterion.

Taking the more conservative BIC, the joint intercept-only model is preferred compared to the individual quadratic model (BIC: 146.6 vs BIC: 160.9). This means that taking the BIC the hypothesis (H.2) *of a frequency dependent variation in sickness sensitivity which also varies across different individuals* is rejected. Based on the AIC however, the individual quadratic model is preferred over the fixed intercept and fixed quadratic models (AIC:31.0 vs 144.2 and 144.0). This is confirmed using a likelihood-ratio test where $p < 0.001$. Therefore, the hypothesis (H.2) *of a frequency dependent variation in sickness sensitivity which also varies across different individuals* is accepted (Table 2).

**Table 2:** Table showing both individual and group fits for 18 participants

Fit type	SSE	Log-likelihood	AIC	BIC
Individual intercept	12.0	− 23.2	82.4	125.7
Individual quadratic	5.0	38.5	31.0	160.9
Joint intercept	27.5	− 71.1	144.2	146.6
Joint quadratic	26.8	− 69.0	144.0	151.2

### Sickness and perception

#### Velocity storage and subjective vertical time constant

As per *H.3, we expected there to be a negative correlation between the frequency sensitivity of sickness and (H.3a) the subjective vertical time constant (as computed simply from the centrifugation results), and (H.3b) the velocity storage time constant*. The spectral centroid was taken as the point of peak sickness. The correlations between this frequency and the perception parameters were computed for those subjects for which both the appropriate perception data and at least four sickness runs existed.

**Fig. 8:**
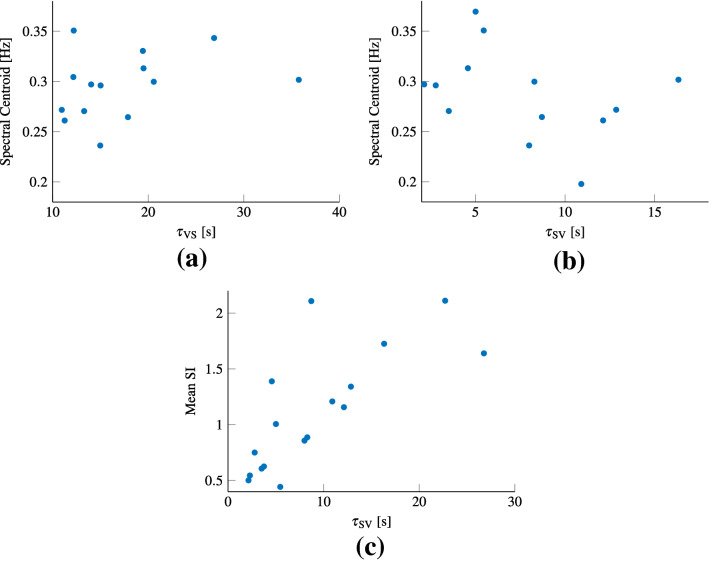
**a** shows the spectral centroid against the velocity storage time constant. **b** shows the spectral centroid against the subjective vertical time constant. **c** Mean sickness sensitivity against the subjective vertical time constant

The correlation coefficients between the spectral centroid and the velocity storage (shown in Fig. 8a) and subjective vertical time constants (shown in Fig. 8b) were *r*(14) = 0.32 (*p* = 0.26) and *r*(13) = − 0.37 (*p* = 0.29), respectively. *Therefore, the evidence does not support the hypothesis of a correlation between the subjective vertical and the velocity storage time constants and the frequency of peak sickness (our H.3a and H.3b).*

#### Cross-over frequency

The mean cross-over frequency of the 14 participants was 0.21 Hz, this ranged from 0.04 Hz to 0.42 Hz. This approximately matched the frequency range where sickness is seen for horizontal accelerations.

For 11 participants both the quadratic function describing the peak sickness frequency and the MSOM fits was estimated. The correlation coefficient between the spectral centroid and the cross-over frequency was *r*(11) = 0.26 (*p* = 0.44). Therefore, *the evidence does not support a correlation between the cross-over frequency and the frequency of peak sickness (H.3a)*.

#### Average sickness sensitivity and perception

In line with earlier literature (Dai et al. 2003, 2007, 2010), motion sickness sensitivity may be correlated with perception parameters. To evaluate this, a mean SI was computed by averaging across all frequency conditions available for each individual participant.

The correlation coefficients between the mean SI and the velocity storage and the subjective vertical time constants were *r*(18) = 0.31 (*p* = 0.21) and *r*(17) = 0.74 (*p* = 0.0006), respectively (the latter is shown in Fig. 8c). For the cross-over frequency this was *r*(14)= − 0.54 (*p* = 0.047). *Thus, our data provides evidence for a relationship between individuals’ overall sickness sensitivity and their subjective vertical time constant (H.3c).*

## Discussion

For the first time, this study investigated individual frequency sensitivity of motion sickness to fore-aft accelerations. Moreover, it analyzes the relationship between individual sensitivity to motion sickness and motion perception. Participants underwent centrifugation and EVAR motions such that perception parameters could be obtained. After this, each participant experienced sickening translational accelerations of varying frequencies in the fore-aft direction. Sickness was quantified for each individual at five different frequencies using an aggregate metric obtained using factor analysis. We then attempted to correlate the obtained individual frequency-sensitivity curves to the perception parameters of the participants. In the following we discuss our findings in relation to the hypotheses and consider methodological issues with quantifying motion sickness and perception.

### Quantifying sickness

Literature shows many ways of quantifying sickness: O’Hanlon (1974) use the motion sickness incidence (MSI), which is the proportion of experiment participants that have vomited before the motion end point. In a similar manner, Donohew and Griffin (2004) use the proportion of those that developed at least mild nausea as a metric and Golding and Markey (1996); Golding et al. (1997) use sickness ratings at motion end point, but also the time taken to reach a particular sickness rating.

The choice of the scale to use is heavily dependent on the experimental design and the aim of the experiment, with each choice presenting advantages and disadvantages. For instance, the MSI is an objective measure of sickness. However, it is only feasible if one wishes to explore the end point of sickness. Using the proportion of the sample to reach a certain level of sickness is good for summarizing group-level sensitivity, but cannot capture individual responses. Moreover, it is only a good measure if a large number of people do reach a certain level of sickness, which is also the case for “time to” measures of sickness sensitivity. In our study only 7 out of 23 unique individuals reached mild nausea, i.e., about a third, compared to over half in the most sickening conditions of Donohew and Griffin (2004). This means that in our experiment not enough people got sufficiently sick for the differences in the percentages to be a meaningful measure. Time-to-sickness rating measures suffer from a similar need of having a large proportion of participants reaching a certain level of sickness. For those participants that do not reach the sickness threshold, the maximum experiment time is set artificially as the time to sickness. This operation distorts the data. Moreover, with the aim of measuring individual frequency sensitivity, there is no way of ensuring an individual will get to the same level of sickness within the available period of time across the frequency spectrum. In an attempt to overcome the limitations of individual metrics, this study uniquely used a joint Sickness Index (SI). The novelty is that the SI is a combination of the MISC, which is a generalized uni-dimensional sickness score that can be obtained quickly and regularly, with parts of the MSAQ, which is more elaborate and can only be completed after an experimental session. By combining these metrics, a more accurate representation of the latent sickness construct may be obtained than when using either metric in isolation.

### Group sickness sensitivity

Looking at the group sensitivity in our study, we found *no significant variation in the SI between the frequency conditions studied, meaning a plateau in the group sensitivity to sickness up to 0.5 Hz.* This finding contrasts with our Hypothesis 1 and earlier literature: for example, Golding and Markey (1996); Golding et al. (1997) found no significant difference in the sickness end point ratings from 0.205 to 0.5 Hz. However, they did find a significant difference in the time-to-sickness rating within this range. However, as discussed above, such a metric could not be sensibly computed for our study. Golding overall argues for a slight decrease in motion sickness sensitivity of − 3 to − 4 dB per octave from 0.2 to 0.4 Hz. This is in disagreement with Donohew and Griffin (2004), who finds a very sharp decrease of −12 db per octave from 0.25–0.8 Hz.

Therefore, the evidence combined over studies seems to indicate a small decrease in group sensitivity from 0.4 Hz to 0.5 Hz, after which there is a sharper decrease. The variability within these studies may be influenced by a large set of factors. One of these is the sickness metric used. For instance, Donohew and Griffin (2004) normalized the percentage reaching mild nausea with the root mean square (RMS) acceleration of their platform, assuming a linear relationship between mild nausea and RMS acceleration magnitude. Another cause can be the large scatter in individual sensitivities. For instance, in Golding and Markey (1996) 8/12 participants reached a sickness rating of 4 for the 0.5 Hz condition, whereas in Golding et al. (1997) only 3/12 did so. This large individual variability is also supported directly by our observations.

### Individual sickness sensitivity

The present study is the first investigation of individual frequency sensitivity of motion sickness to fore-aft accelerations. We find that although the group sensitivity is flat, individual sensitivities vary steeply, with some participants more susceptible to low-frequency oscillations, and others more susceptible to mid- and high-frequency accelerations. To test for individual variability in the frequency sensitivities, we compared the fit of quadratic models to the fit of intercept-only models on the basis of the AIC and BIC model fit scores. The AIC values supported the conclusion that there is frequency sensitivity in motion sickness responses and that the peak sensitivity varies between individuals, and this was confirmed using the likelihood-ratio test ($p < 0.001$). The BIC values supported a conclusion that there is individual variability, but only with respect to the overall sensitivity; using this score, there was no evidence for frequency sensitivity. A reason for the disagreement between the BIC and AIC scores is that the BIC is much more conservative. Here, the penalisation of model complexity scales with the logarithm of the number of observations, which for our case was 82. Moreover, a likely cause for the favorability of the intercept-only model according to the BIC score is that some participants did not get sick (due to the low acceleration amplitude) and so had a flatter frequency sensitivity. Likewise, for some that did get sick, the range of frequencies we observed may not have been large enough to measure the attenuation of their sickness sensitivity. Moreover, as motion sickness is not experienced at very low (i.e $\le$0.03 Hz, Donohew and Griffin (2004)) or high (i.e., $\ge$ 0.5 Hz frequencies, Donohew and Griffin (2004); Cheung and Nakashima (2006)), the BIC favored intercept-only model will not be tenable when a larger frequency range is considered. Therefore, the AIC-favored Individual Quadratic model is more credible, supporting that there are indeed differences in the motion sickness frequency sensitivity of our participants. Literature (Miller and Graybiel 1969; Irmak et al. 2020) shows that individuals exhibit repeatable sickness response when subject to the same motion stimuli over consecutive motion trials, while our results show large individual variation indicating an effect of frequency sensitivity. Consequently, we accept the hypothesis that *(H.2) The frequency-dependent sickness sensitivity varies across different individuals.* This also means that individual sickness frequency sensitivities are not represented well by the group-level frequency sensitivity.

### Perception and sickness

As summarised in the introduction, there have been several studies linking motion perception parameters to motion sickness. However, to the authors’ knowledge none have taken the frequency-domain approach usually employed in engineering disciplines to resolve the problem of how the two may be mechanistically related. Based on our review of the literature, we hypothesized that motion sickness frequency sensitivity may be related to three motion perception parameters: the velocity storage time constant, the subjective vertical time constant (from simple centrifugation) and the cross-over frequency as per the MSOM.

The subjective vertical time constant that was computed was similar to values reported in the literature (see Fig. 5a). We note that the end value of the perceived tilt angle with respect to the GIF could be different had the participants been facing towards the centre of rotation. Here, the added utricular shear in the hyper-gravity environment of the centrifuge would have caused an over-estimation of roll tilt and a larger difference with the GIF (Clark et al. 2015). However, this is not likely to affect the time dynamics. The velocity storage time constant was also similar to values reported in the literature (see Fig. 5b). For the subjective angular velocity perception we report plateauing of the perceived velocity for 11 participants. This is in line with Bertolini et al. (2011), who found the same plateau for 10 of their participants and a faster decay for the remaining 6.

Overall, both the recorded tilt perception and angular velocity perception results match previous literature findings and are therefore valid correlates of the internal state estimates. Moreover, they display substantial variation between participants (Fig. 5) indicating a positive perspective to explain individual motion sickness sensitivity.

Our results showed that *the subjective vertical time constant was negatively correlated with peak sickness frequency whereas the cross-over frequency (H.3a)* and *the velocity storage time constant were positively correlated (H.3b)*. However, the correlations were weak, and did not reach statistical significance. It is possible that this was due to a lack of statistical power. Due to the corona virus pandemic human-subject experiments were restricted during the course of our study, which unfortunately led to some missing frequencies in some of our participants. Despite the correlations not reaching significance, we can argue their implications. For instance, the positive correlation between the cross-over frequency and the frequency sensitivity is in line with the hypothesis that sickness occurs at the point of most perceptual ambiguity. The subjective vertical time constant as determined from centrifugation is inversely proportional to this cross-over frequency, which explains its negative correlation with sickness sensitivity. Moreover, the range of cross-over frequencies obtained from MSOM fits are within the range where we find maximum sickness to occur. Although our findings do not provide irrefutable proof, they are consistent with a relation between gravito-inertial ambiguity resolution and motion sickness induced by translational accelerations.

A key finding is the *(H.3c) strong correlation (r = 0.74) between an individual’s average motion sickness sensitivity and their subjective vertical time constant.* To confirm this wasn’t just due to the complex determination of the overall sickness using the mean SI we also computed the correlation between mean MISC and the subjective vertical time constant, this was *r*(17) = 0.8 (*p* = 0.0001). To the authors’ knowledge, such a correlation has not been reported in the literature. Literature does, however, report on the importance of the velocity storage time constant as a marker for sensitivity (Dai et al. 2003, 2007). In our experiment this correlation was not evident (*r* = 0.32). The likely reason for this is the absence of any rotational stimuli in our experimental paradigm that would contribute to sickness. In studies that did find a correlation with the velocity storage time constant, rotational motions were used to induce sickness, namely, the cross-coupled coriolis stimulation. Our results thus suggest that for purely translational stimuli, the subjective vertical time constant is a major determinant of sensitivity.

To summarize, the time constant of the subjective vertical is positively correlated with the overall sickness sensitivity. It is also known from literature that the subjective vertical time constant is determined by the frequency properties of gravito-inertial ambiguity resolution (Laurens and Angelaki 2011). Moreover, we know from our study that individuals have substantial variance in their frequency sensitivities. However, contrary to our initial expectation, we did not find a correlation between the subjective vertical time constant and the frequency sensitivity of individuals that might explain this variance. Given what is known about the relevance of the subjective vertical time to spatial orientation and, (as this study also finds) to general sickness sensitivity, this is surprising. Hence, further investigations should be devoted to the subjective vertical time constant and the cross-over frequency. One way to do this is, just as Dai et al. (2003) had done for the velocity storage time constant, is by studying the effect of motion sickness habituation on the subjective vertical time constant. Another way is by explicitly attempting to measure the cross-over frequency and comparing the sickness seen here with adjacent frequencies.

### Limitations

The statistical analyses in our paper provided limited support for conclusions on frequency sensitivity. The reason for this is twofold. As discussed above, one part of the issue is the choice of a statistical criterion (AIC vs BIC). Since there is no single true or correct way to resolve this, we must rely on theoretical considerations Dziak et al. (2019). A second issue, which arguably affects any study on motion sickness, is that there was only a limited number of observations available and that these observations are to some extent corrupted by noise, i.e., the signal-to-noise ratio. The range of frequencies tested may not have been large enough to capture the peak sensitivity for all individuals. The thresholding at 0.15 Hz means that the spectral centroid can never be less than 0.15 Hz. This has important implications for the correlations between the spectral centroid and the self-motion parameters. Participants who were sensitive to lower frequencies and likely had even lower spectral-centroids were biased towards higher values, therefore, potentially reducing the significance of the correlation we found between the spectral centroid and the self-motion parameters.

The choice of frequencies was a compromise between, on the one hand, the range of frequencies where motion sickness may peak according to the literature and the minimum amplitude required to induce sickness, and on the other hand the limitations of the simulator. To increase the signal-to-noise ratio as well as reduce bias, future studies may test a broader range of frequencies and/or increase the motion amplitude.

Statistical power can be enhanced increasing the number of repetitions. However, this presents logistical and dropout related difficulties associated with getting the same participant to become sick on a weekly basis for multiple months. To illustrate, in the present experiment, it took more than a month of continuous testing to obtain a single data point for five frequencies for a sample of 23 participants. Each of these participants was required to return to the experimental facility six times (including the experimental session for collection of perception data). Simply collecting one additional repetition for each experimental condition requires nearly twice that amount of time for data collection and the number of visits for each participant.

In future studies, it may also be possible to increase the signal-to-noise ratio by collecting and merging different kinds of data. In the present study, we did this by combining the MISC and MSAQ data into a Sickness Index. Similarly, we recorded eye-movements with the intent to combine this data with the perception data to improve the state-estimation, but these recordings unfortunately were not of sufficient quality for further analysis. Finally, the cross-over frequency was not directly measured but inferred from the MSOM. There may be inaccuracies introduced by fitting the model; measuring the cross-over frequency directly by adopting a different experimental paradigm may also contribute to our understanding of its relation to motion sickness.

This study was designed to relate motion sickness to motion perception and did not investigate postural instability (Riccio and Stoffregen 1991). Future studies could in particular relate the subjective vertical time constant to postural stabilisation through experiments and models of sensory integration, postural stabilisation and motion sickness development.

## Conclusion

This study investigated individual frequency sensitivity of motion sickness to fore-aft accelerations and its relationship to individual parameters of motion perception. We found that at a group level, sickness sensitivity was frequency invariant from 0.15-0.5 Hz. Importantly we found support for differing frequency sensitivity to motion sickness between individuals, with some being susceptible to low-frequency motions, others to intermediate and others to high-frequency accelerations. Therefore, group sensitivity does not represent individual sensitivities. We observed no significant correlations between the velocity storage time constant, the subjective vertical time constant and cross-over frequency and motion sickness frequency sensitivity. This may be due to the limited number of observations. The direction of the effects, however, does support the notion that the cross-over frequency, which is the point of maximum perceptual ambiguity between acceleration and gravity perception, is indicative of the frequency at which the sickness response has its maximum. Moreover, we observed a strong correlation (*r* = 0.74) between the subjective vertical time constant and overall sickness sensitivity. This may be indicative of the importance of verticality perception to sickness development during exposure to translational sickness stimuli.

These findings are of particular significance to motion sickness modelling and indicate that etiologically valid models should fit individual, rather than group-level, frequency sensitivities. Additionally, our results indicate that future models should take into account the apparent relationship between the subjective vertical time constant and the overall motion sickness sensitivity. Lastly, the results are of particular significance to the automotive community, as they highlight the individual nature of motion sickness and the need for caution when using group sickness sensitivities to tune vehicle controllers for the reduction of motion sickness.

## Supplementary Information

Below is the link to the electronic supplementary material.

Supplementary material 1 (PDF 468 kb)

Supplementary material 1 (CSV 6 kb)
