# Supplementary material for: Individual motion perception parameters and motion sickness frequency sensitivity in fore-aft motion
Source: Exp Brain Res. 2021 Mar 29;239(6):1727–45. doi: 10.1007/s00221-021-06093-w (PMC8006642; doi:10.1007/s00221-021-06093-w)
Supplement: Supplementary file 1 — Supplementary material 1 (PDF 468 kb) [file 221_2021_6093_MOESM1_ESM.pdf]

Individual motion perception parameters and motion sickness frequency sensitivity in fore-aft motion – Appendix

T. Irmak, K.N. de Winkel, D. Pool, H.H. Bülthoff, R. Happee

Figure 9 shows the centrifugation and EVAR responses of each participant for whom data could be collected. Figure 9a shows the first 60 s of the centrifugation response as indicated by participants using the angular pitch of a metal rod connected to a potentiometer. Figure 9a shows the entire 120 s of the EVAR response indicated by rotating the rod away from the body or towards the body, depending on the perceived direction and magnitude of angular velocity in yaw. The y-axis has been normalized to the maximum displacement of the rod.

T.Irmak
Delft University of Technology
Mekelweg 2
2628 CD Delft South Holland, Netherlands
E-mail: t.irmak@tudelft.nl

K.N. de Winkel
Delft University of Technology
Mekelweg 2
2628 CD Delft South Holland, Netherlands
E-mail: K.N.deWinkel@tudelft.nl
ORCID: 0000-0003-0534-2723

D.M. Pool
Delft University of Technology
Kluyverweg 1
2629 HS Delft South Holland, Netherlands
E-mail: D.M.Pool@tudelft.nl

Heinrich H. Bülthoff
Max Planck Institute for Biological Cybernetics
Max-Planck-Ring 14
72076, Tübingen Baden-Württemberg, Germany Tel.: +49-7071-601201
Fax: +49-7071-601616
E-mail: heinrich.buelthoff@tuebingen.mpg.de
ORCID: 0000-0003-2568-0607

R. Happee
Delft University of Technology
Mekelweg 2
2628 CD Delft South Holland, Netherlands
E-mail: R.Happee@tudelft.nl

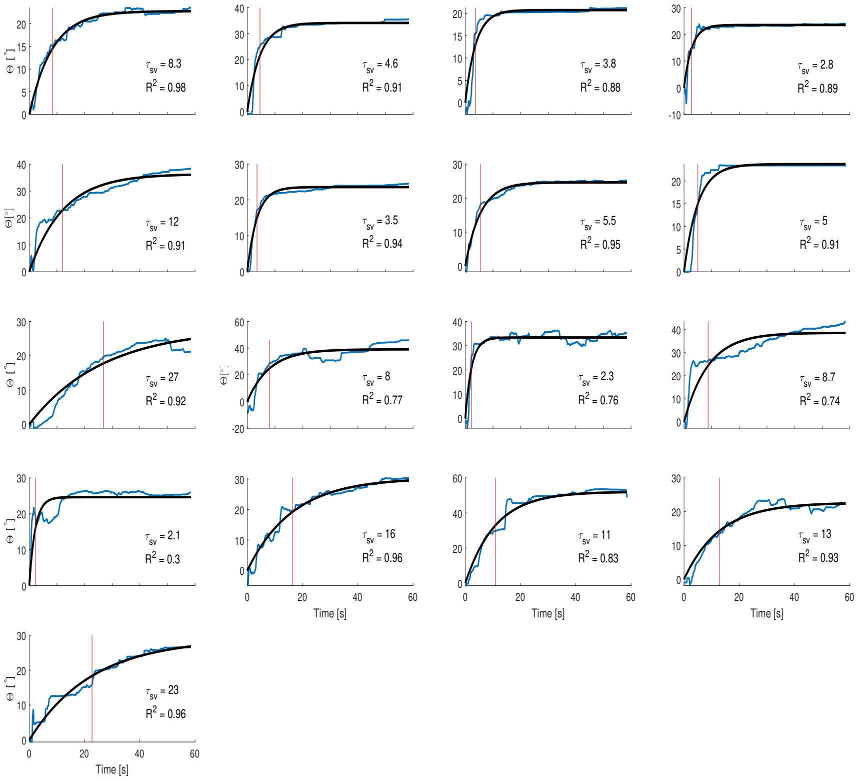

(a) Centrifugation

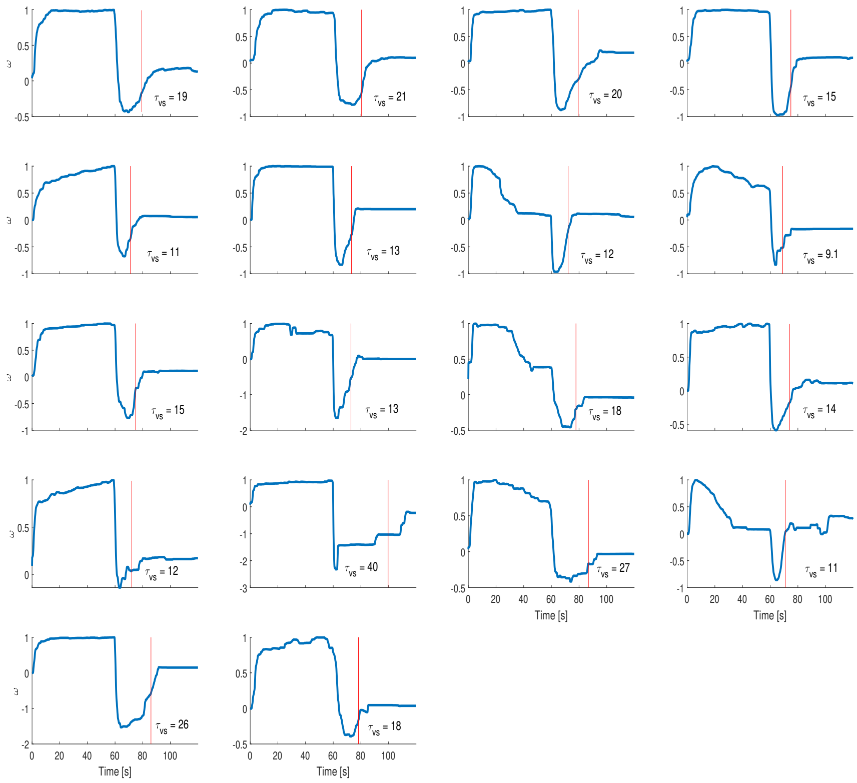

(b) EVAR

Fig. 9 Perceptual responses for all individuals averaged over repetitions. a is the subjective vertical tilt in centrifugation. At 0 s the centrifugation is started and maintained until 60 s after which it is stopped. At 120 s another trial is initiated. The black line shows the fitted exponential model, the R^2 value and the time constant τ_{sv} is also shown in the text box. The vertical red line shows this time constant on the plot itself. b shows the angular velocity after rotation in EVAR. At 0 s the EVAR motion of the simulator is stopped and at 60 s a new trial is initiated. The vertical red line shows the point at which perceived normalized angular velocity decays to 63.2% of the way down to steady state

Figure 10 shows the fits of the MSOM to the centrifugation and EVAR aftereffect responses shown above. Fits were only performed on those participants for whom both EVAR and centrifugation were collected. Figure 10a shows the fits for centrifugation that are, in most cases, a good fit to the experimental data. Figure 10b shows the fits for EVAR that doe not accurately predict the plateauing effect seen in the experimental data. For this a more complex model would have to be introduced.

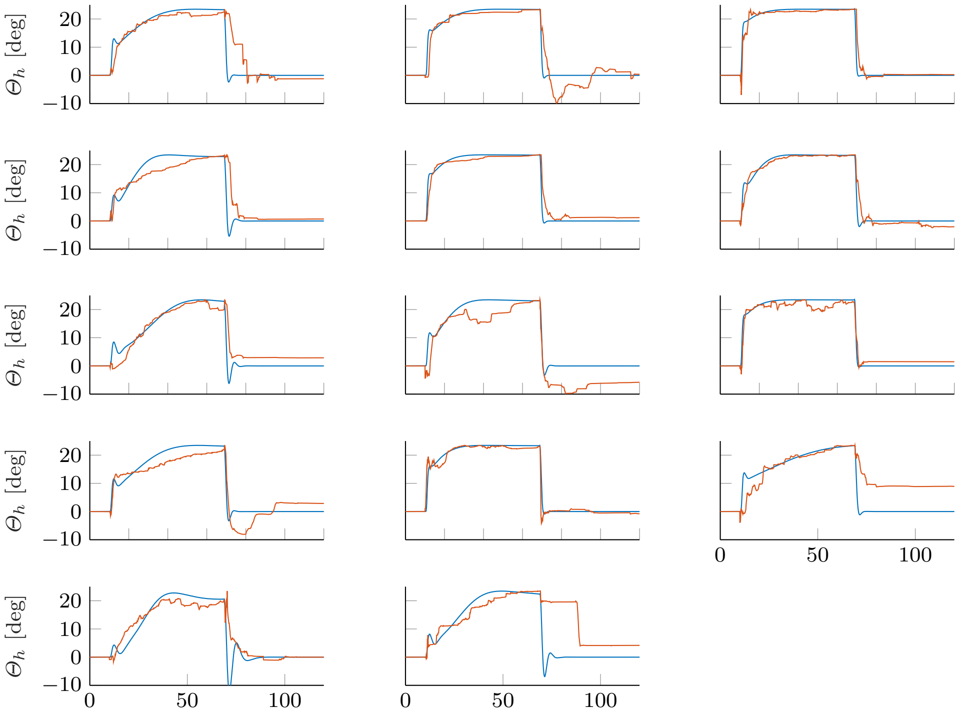

(a) Perceived subjective vertical tilt.

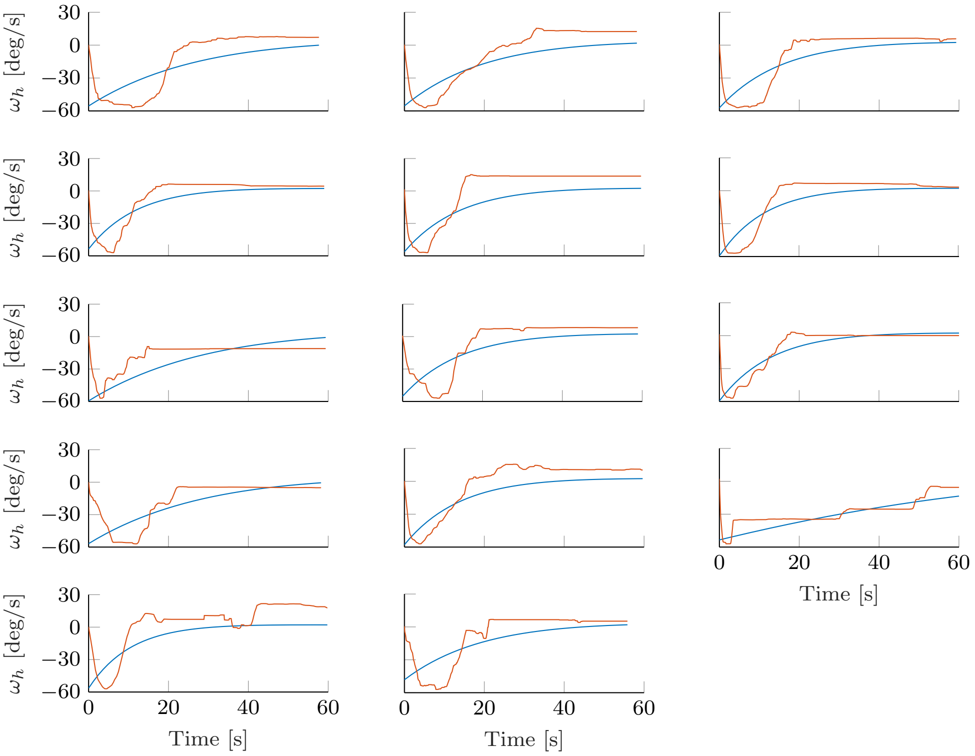

(b) Perceived angular velocity.

Fig. 10 MSOM fits for all individuals for which both EVAR and centrifugation data exists. *Top* are fits for the subjective vertical tilt for each participant. *Bottom* are fits for the perceived angular velocity after rotation. Only the point after the zero crossing for EVAR was used for the error minimization

Figure 11 shows the gain of acceleration and gravity estimation with respect to frequency, as computed from the MSOM. At low frequencies the gravity estimate has the highest gain, which then crosses over at an intermediate frequency, after which the acceleration gain increases. The point where the curves cross is, by definition, the cross-over frequency.

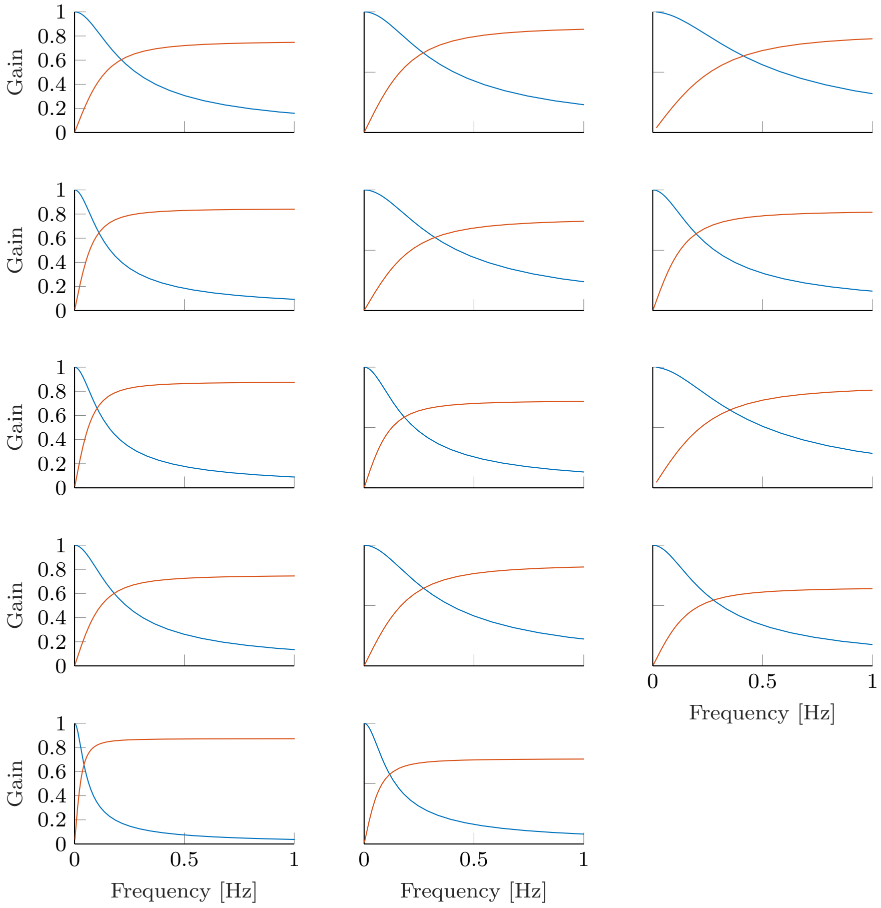

Fig. 11 Gravity and acceleration perception frequency responses as obtained from one of the runs of MSOM

Figure 12 shows the MISC as a function of time for all participants and for all frequency conditions. The MISC rating was queried in 30 s intervals. Here, sickness responses are generally seen to converge to a value below the cut off value of MISC 6.

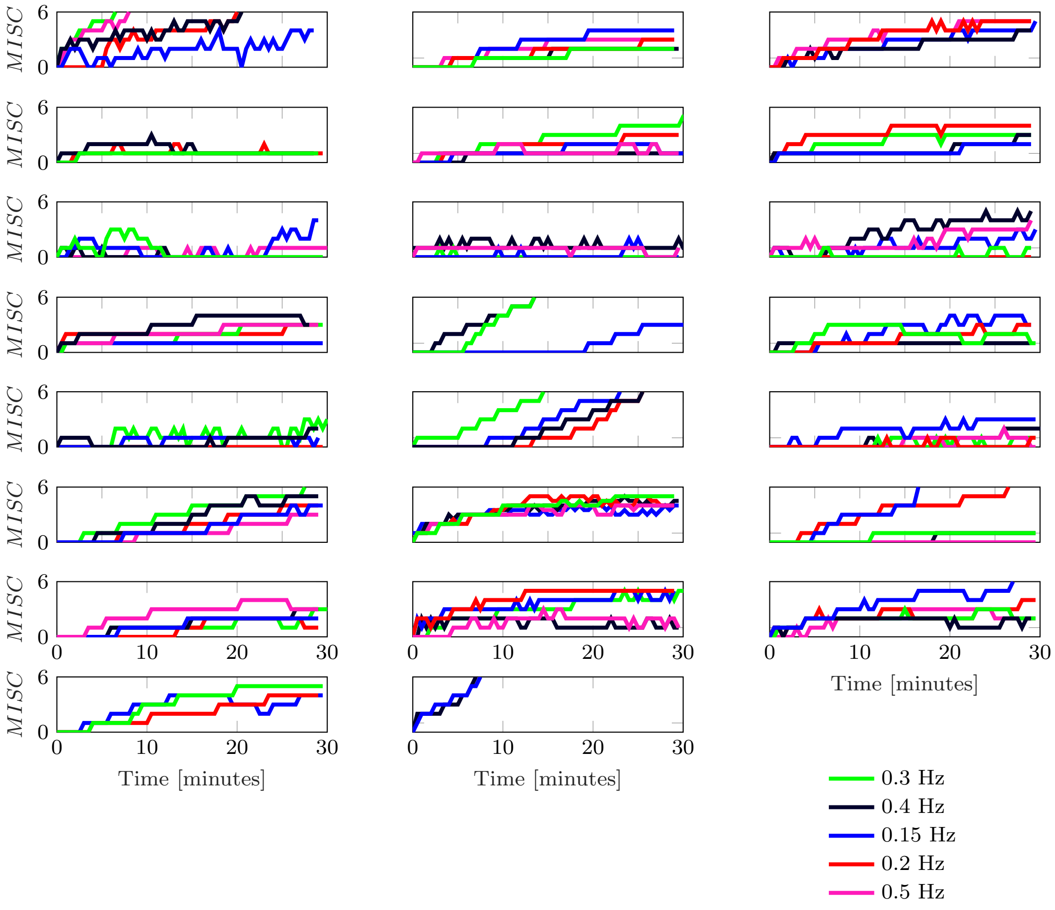

Fig. 12 MISC Trajectories for all individuals

Figure 13 shows the residuals of the fitted Fixed Effect Quadratic model. An ideal model fit would have residuals centered on zero with limited variance for each participant. Here the residuals for each participant are centred appreciably above or below zero, with large variances, indicating a poor fit with the Fixed Effect Quadratic model.

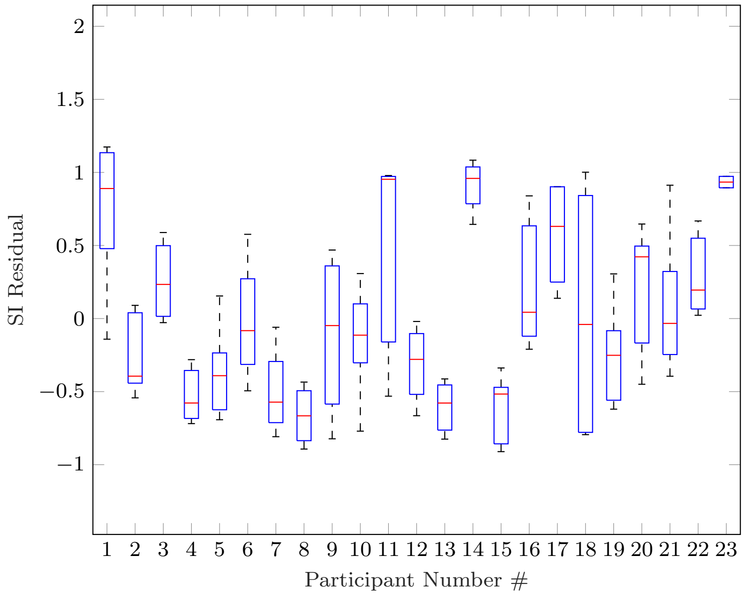

Fig. 13 Box plot of the residuals for our fitted Fixed Effect Quadratic model. Residuals are, on average, mostly above or below zero, indicating that the model has failed to account for subject-specific effects
